# *CYP11B1* variants influence skeletal maturation via alternative splicing

**DOI:** 10.1038/s42003-021-02774-y

**Published:** 2021-11-09

**Authors:** Olja Grgic, Matthew R. Gazzara, Alessandra Chesi, Carolina Medina-Gomez, Diana L. Cousminer, Jonathan A. Mitchell, Vid Prijatelj, Jard de Vries, Enisa Shevroja, Shana E. McCormack, Heidi J. Kalkwarf, Joan M. Lappe, Vicente Gilsanz, Sharon E. Oberfield, John A. Shepherd, Andrea Kelly, Soroosh Mahboubi, Fabio R. Faucz, Richard A. Feelders, Frank H. de Jong, Andre G. Uitterlinden, Jenny A. Visser, Louis R. Ghanem, Eppo B. Wolvius, Leo J. Hofland, Constantine A. Stratakis, Babette S. Zemel, Yoseph Barash, Struan F. A. Grant, Fernando Rivadeneira

**Affiliations:** 1 Department of Internal Medicine, Erasmus MC, University Medical Center Rotterdam, Dr Molewaterplein 40, 3015 GD Rotterdam, The Netherlands; 2 Department of Oral and Maxillofacial Surgery, Erasmus MC, University Medical Center Rotterdam, Dr Molewaterplein 40, 3015 GD Rotterdam, The Netherlands; 3 The Generation R Study, Erasmus MC, University Medical Center Rotterdam, Dr Molewaterplein 40, 3015 GD Rotterdam, The Netherlands; 4 Department of Genetics, Perelman School of Medicine, University of Pennsylvania, 2615 Civic Center Boulevard, Philadelphia, PA 19104 USA; 5 Department of Biochemistry and Biophysics, Perelman School of Medicine, University of Pennsylvania, 2615 Civic Center Boulevard, Philadelphia, PA 19104 USA; 6 Center for Spatial and Functional Genomics, Children’s Hospital of Philadelphia, 3401 Civic Center Boulevard, Philadelphia, PA 19104 USA; 7 Department of Epidemiology, Erasmus MC, University Medical Center Rotterdam, Dr Molewaterplein 40, 3015 GD Rotterdam, The Netherlands; 8 Division of Human Genetics, Children’s Hospital of Philadelphia, 3401 Civic Center Boulevard, Philadelphia, PA 19104 USA; 9 Department of Pediatrics, Perelman School of Medicine, University of Pennsylvania, 3400 Civic Center Boulevard, Philadelphia, PA 19104 USA; 10 Division of Gastroenterology, Hepatology, and Nutrition, The Children’s Hospital of Philadelphia, 3401 Civic Center Boulevard Philadelphia, Philadelphia, PA 19104 USA; 11 Bone and Joint Department, Center of Bone Diseases, Lausanne University Hospital, Rue du Bugnon 46, 1011 Lausanne, Switzerland; 12 Division of Endocrinology and Diabetes, Children’s Hospital of Philadelphia, 3401 Civic Center Boulevard Philadelphia, Philadelphia, PA 19104 USA; 13 Department of Pediatrics, Cincinnati Children’s Hospital Medical Center, University of Cincinnati College of Medicine, 3333 Burnet Ave, Cincinnati, OH 45229 USA; 14 Division of Endocrinology, Creighton University, 2500 California Plaza, Omaha, NE 68178 USA; 15 Division of Orthopedic Surgery, Children’s Hospital Los Angeles, Keck School of Medicine, University of Southern California, 1975 Zonal Ave, Los Angeles, CA 90033 USA; 16 Department of Radiology, Children’s Hospital Los Angeles, Keck School of Medicine, University of Southern California, 1975 Zonal Ave, Los Angeles, CA 90033 USA; 17 Division of Pediatric Endocrinology, Morgan Stanley Children’s Hospital, Columbia University Irving Medical Center, 622 West 168th Street, PH17 W 307, New York, NY 10032 USA; 18 Cancer Epidemiology, University of Hawai’i Cancer Center, 701 Ilalo St, Honolulu, HI 96813 USA; 19 Department of Radiology, Children’s Hospital of Philadelphia, 3401 Civic Center Boulevard, Philadelphia, PA 19104 USA; 20 Section on Endocrinology and Genetics, Eunice Kennedy Shriver National Institute of Child Health and Human Development (NICHD), National Institutes of Health, 6710 Rockledge Dr, Bethesda, MD 20817 USA

**Keywords:** Gene regulation, Bone development

## Abstract

We performed genome-wide association study meta-analysis to identify genetic determinants of skeletal age (SA) deviating in multiple growth disorders. The joint meta-analysis (N = 4557) in two multiethnic cohorts of school-aged children identified one locus, *CYP11B1* (expression confined to the adrenal gland), robustly associated with SA (rs6471570-A; β = 0.14; *P* = 6.2 × 10^−12^). rs6410 (a synonymous variant in the first exon of *CYP11B1* in high LD with rs6471570), was prioritized for functional follow-up being second most significant and the one closest to the first intron-exon boundary. In 208 adrenal RNA-seq samples from GTEx, C-allele of rs6410 was associated with intron 3 retention (*P* = 8.11 × 10^−40^), exon 4 inclusion (*P* = 4.29 × 10^−34^), and decreased exon 3 and 5 splicing (*P* = 7.85 × 10^−43^), replicated using RT-PCR in 15 adrenal samples. As *CYP11B1* encodes 11-β-hydroxylase, involved in adrenal glucocorticoid and mineralocorticoid biosynthesis, our findings highlight the role of adrenal steroidogenesis in SA in healthy children, suggesting alternative splicing as a likely underlying mechanism.

## Abstract (web summary)

Olja Grgic, Matthew Gazzara, and Alessandra Chesi et al. perform a genome-wide association study meta-analysis for skeletal age in two pediatric cohorts. They observe that variation in the adrenal gene, *CYP11B1*, impacts its alternative splicing, suggesting that adrenal steroidogenesis may contribute toward variation in skeletal age in otherwise healthy children.

## Introduction

Skeletal age (SA) is a maturational process in which the child’s skeleton increases in shape, size, and density throughout adolescence until achieving the adult stage^1^. SA is subject to the influence of behavioral, environmental, and genetic factors^2^. Overall, girls present on average higher SA as compared to boys, and children of African ethnic background as compared to non-Africans^1,3^.

Different disorders resulting in impairments of skeletal maturation and often associated with short stature are caused by loss of function mutations in diverse genes. Genes in the Online Mendelian Inheritance in Man (OMIM) database (https://www.omim.org)^4^ include *ACAN* (SSOAD; MIM#165800), *CSGALNACT1* (SDJLABA; MIM#618870), *NFIX* (MRSHSS; MIM#602535), *EZH2* (WVS, MIM#277590), *PTHR1* (BOCD; MIM#215045), *PTDSS1* (LMHD; MIM#151050), *KIP2* (BWS; MIM#130650), *NSD1* (SOTOS1; MIM#117550), *SRY* (Kennerknecht Syndrome; MIM600908) and *CYP11B1* (CAH; MIM#202010). Among these, congenital adrenal hyperplasia (CAH; MIM#202010) refers to a group of disorders that arise from defective steroidogenesis, resulting in the accumulation of the steroid precursors. Clinical consequences include adrenal insufficiency, genital ambiguity or alterations of sex development, infertility, short stature, hypertension, and increased risk of metabolic syndrome during adolescence and adulthood. The severity and clinical features of CAH depend on the severity of the enzymatic defect, which can occur in any of the five steroidogenic enzymes: cytochrome P450 side-chain cleavage enzyme encoded by *CYP11A1*; 21-α-hydroxylase encoded by *CYP21A2*; 3β-hydroxysteroid dehydrogenase 2 encoded by *HSD3B2*; 17-hydroxylase/17, 20-lyase encoded by *CYP17A1* and 11-β-hydroxylase encoded by the *CYP11B1*^5^.

Hence, SA is considered a clinically relevant parameter that is crucial for the assessment of health and wellbeing (e.g., constructing growth charts) or of different medical conditions in children^2,6^. Children observe normal variation in growth, ranging in between advanced or delayed bone age, where a bone age that deviates more than two standard deviations from the mean of a certain age is likely due to a pathologic condition^7^. Further understanding of the mechanisms by which SA is involved in health and disease processes may provide strategies to ensure healthy growth, development, and optimal peak bone mass acquisition^1,8^.

To the best of our knowledge genetic control of SA has never been investigated at the population level using genome-wide association study (GWAS). A GWAS of SA will provide further insight into the underlying biology of human skeletal maturation and associated disorders of short stature. Therefore, we meta-analyzed our two unpublished GWAS of SA in children of school age.

## Results

### Meta-analysis

**Fig. 1:**
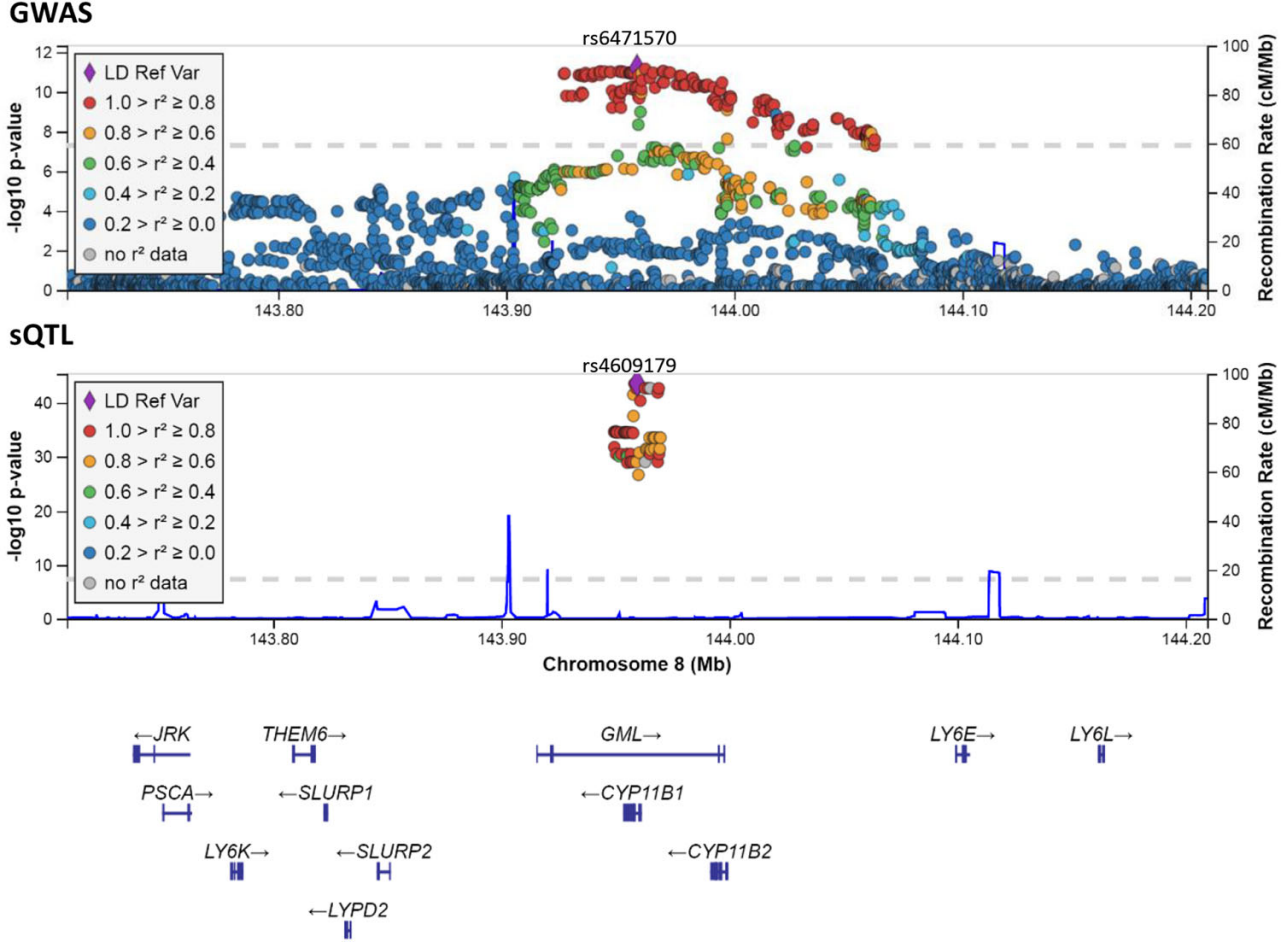
Regional plot for the CYP11B1 locus presented for GWAS and sQTL findings. The most significant variant in the locus is presented as a purple diamond and the flanking variants as circles in different colors according to the level of their linkage disequilibrium (LD) with the lead variant in the CEU-population 1000 genomes. *X* axis shows all the genes in the region between 8:143707405 and 8:144207405. *Y* axis presents the –log10 (*P* value). Source data are available in Supplementary Data 5 (sQTL locus zoom; http://locuszoom.org) or publicly available at the GEFOS website (https://www.gefos.org/) (GWAS locus zoom; http://locuszoom.org).

A GWAS meta-analysis including 9,806,907 single-nucleotide polymorphisms (SNPs) was performed in 4557 children of multiethnic background participating in the Generation R Study (*N* = 3510, 49% boys; mean age 9.8, SD = 0.33 years) and the Bone Mineral Density in Childhood Study (BMDCS) (*N* = 1047, 47% boys; mean age 10.9, SD = 1.39 years) to identify loci associated with SA (assessed in years in both cohorts). Sex-stratified analyses were performed in boys (*N* = 2213) and girls (*N* = 2344). SA was assessed in all children on hand radiographs (BMDCS) or hand DXA scans (Generation R Study; GE-Lunar iDXA) using the Greulich and Pyle Atlas method. Heritability of SA was estimated at 51% in the European subpopulation of children from the Generation R Study (SE = 0.19, *P* = 0.004) (see “Methods”). The characteristics of the study populations are shown in Table 1. QQ plots did not show early inflation of the test statistics in the joint or sex-stratified meta-analyses (*λ*_joint_ = 0.98, Supplementary Fig. 1; *λ*_boys_ = 1.00 and *λ*_girls_ = 0.99, Supplementary Fig. 2). The joint meta-analysis identified variants mapping to the *CYP11B1* locus on chr 8 associated at a genome-wide significant (GWS) level with advanced SA (lead SNP rs6471570-A; MAF = 0.41; *β* = 0.14; *P* = 6.2 × 10^−12^) (Fig. 1, Supplementary Fig. 1 and Supplementary Data 1). Conditional analysis using genome-wide complex trait analysis (GCTA) revealed no secondary signals in this locus (*P* = 5 × 10^−8^). FINEMAP approach^9^ created a credible set of 77 variants (out of which 28 had log10 Bayes factor ≥ 2)^10^. Lead SNP (rs6471570) and rs6410 (the second most significant SNP in our meta-analysis; *P* = 7.0 × 10^−12^) showed the highest log10 Bayes factors of 2.3 and 2.2, respectively, providing considerable evidence for causality (Supplementary Data 2). The polygenic architecture of SA is suggested by four other loci associated with SA at a genome-wide suggestive level, namely at 9q21.32 (*LOC101927575*; rs1030856-A: MAF = 0.29; *β* = 0.12; *P* = 3.5 × 10^−7^); 12q23.3 (*RIC8B*; rs76918979-C: MAF = 0.04; *β* = −0.30; *P* = 1.3 × 10^−7^); 12p13.2 (*BORCS5*; rs7961296-T: MAF = 0.04; *β* = −0.27; *P* = 2.8 × 10^−7^); and in 16q24.3 (*FANCA*; rs148559047-A: MAF = 0.03; *β* = −0.32; *P* = 5.7 × 10^−7^) (Supplementary Data 3). The sex-stratified meta-analyses (Supplementary Data 4) showed nominally significant sex-heterogeneity (*P*_het_ = 0.02) for the lead SNP (rs6471570) in the *CYP11B1* locus in boys (*β* = 0.20; *P* = 1.7 × 10^−10^) and girls (*β* = 0.10; *P* = 0.001).

**Table 1:** Baseline characteristics of participants in the Generation R Study and BMDCS.

Generation R Study				
	*N* (3510) Mean (SD)	Boys (49%) Mean (SD)	Girls (51%) Mean (SD)	*P* ΔSex
Age (years)	9.80 (0.33)	9.80 (0.35)	9.78 (0.31)	0.05
Height (m)	1.42 (0.07)	1.42 (0.06)	1.41 (0.07)	0.04
*Z*_Height	−0.07 (1.00)	−0.05 (0.94)	−0.09 (1.05)	0.25
BMI (kg/m²)	17.54 (2.71)	17.41 (2.55)	17.66 (2.85)	0.01
*Z*_BMI	0.27 (1.03)	0.30 (1.03)	0.24 (1.04)	0.05
Skeletal age (years)	9.42 (1.29)	9.27 (1.49)	9.56 (1.05)	<0.001

BMDCS				
	*N* (1047) Mean (SD)	Boys (47%) Mean (SD)	Girls (53%) Mean (SD)	*P* ΔSex
Age (years)	10.91 (1.39)	10.83 (1.37)	10.98 (1.40)	0.10
Height (m)	1.45 (0.10)	1.44 (0.10)	1.46 (0.10)	0.01
*Z*_Height	0.21 (0.81)	0.16 (0.79)	0.25 (0.83)	0.07
BMI (kg/m^2^)	18.47 (2.76)	18.17 (2.55)	18.73 (2.91)	<0.001
*Z*_BMI	0.25 (0.89)	0.23 (0.90)	0.27 (0.89)	0.44
Skeletal age (years)	10.83 (1.94)	10.48 (1.85)	11.13 (1.97)	<0.001

*N* sample size, *SD* standard deviation, *Z scores standardized values.

### Gene expression

In the Genotype-Tissue Expression project (GTEx v8)^11^, *CYP11B1* expression was largely untraceable across tissues except for the adrenal gland, cervix–ectocervix, and testis. After performing two pair-wise differential expression analyses, it was observed that comparative expression of *CYP11B1* was largely confined to the adrenal gland (Supplementary Fig. 3); with log_2_ fold-change of 13.6 compared to the cervix−ectocervix and 10.4 compared to the testis (both with *P* < 1 × 10^−56^). Lastly, *CYP11B1* was not expressed in bulk bone tissue assessed with RNA-Seq (see “Methods”), with profiles showing complete absence of raw transcript counts in 107 out of 121 available bone samples.

### Splicing analysis of *CYP11B1* variants in GTEx samples

Several of the variants underlying the GWAS signal are in proximity to the intron−exon boundary of *CYP11B1* implying a potential role for *alternative splicing*, a powerful mechanism expanding the regulatory and functional repertoire of genes^12^. To screen *CYP11B1* for splicing quantitative trait loci (sQTL), we used GTEx data (v8) from 208 adrenal gland RNA-seq samples that passed quality control metrics and with whole-genome sequencing data available (see “Methods”). We focused the sQTL analysis on rs6410 (a synonymous variant in the first exon), considering it was: in high LD (*r*^2^ = 0.83) with rs6471570 (the top associated variant); the second most significant variant in our meta-analysis (*P* = 7.0 × 10^−12^); with the second highest log10 Bayes factor in FINEMAP approach; and is located within 15 nucleotides of the first intron−exon boundary of *CYP11B1*. The rs6410 variant explained 1.06% of the variation in SA. In the Generation R Study (*N* = 3510; see “Methods: Statistics and reproducibility”) TT carriers of rs6410 had on average 5 (SE = 0.06) months higher SA as compared to CC carriers (*P* < 0.0001) (least square means corrected for age, sex, BMI and ten genomic PCs used). Supplementary Fig. 4 demonstrates differences in raw (relative) SA across genotypes. We hypothesized that rs6410 is associated with changes in the expression or isoform usage of *CYP11B1*. Surveying rs6410 and variants in its proximity, in order to capture other potentially causal variants in LD with the candidate SNP (see “Methods”), we found several strong associations with splicing variations (sQTLs), meaning sQTL variants affected the relative abundance of transcripts and, potentially, protein product isoforms. Given the sex-heterogeneity of the GWAS signal, we performed sex-stratified sQTLs analyses in males (*N* = 122) and females (*N* = 86).

RNA-seq reads were mapped to the hg38 reference genome using STAR-2.5.3a^13^, and inclusion levels of alternative splice junctions were quantified using the *Modeling Alternative Junction Inclusion Quantification* (MAJIQ v.2.1)^14,15^ algorithm followed by an sQTL pipeline (see “Methods”)^16^. Briefly, MAJIQ defines and quantifies local splicing variations (LSV) from RNA-seq data. LSVs are defined by splits in a splice graph that correspond to alternative splice junctions and/or retained introns and reflect the splicing choices available during the production of the final transcripts of a gene^14,15^. MAJIQ quantifies the relative usage of each of these splice junctions or intron retentions within an LSV as Percent Selected Index (PSI or Ψ), where PSI for an individual junction or intron corresponds to the percent of transcripts that made that splicing choice. The sQTL pipeline tests for a significant association between a given SNP and PSI values for samples stratified by genotype (see “Methods”). Additionally, we required a minimum change in inclusion of at least 10% (|ΔPSI| > 10%) when comparing the expected PSI values of homozygous reference and homozygous alternative genotypes.

**Fig. 2:**
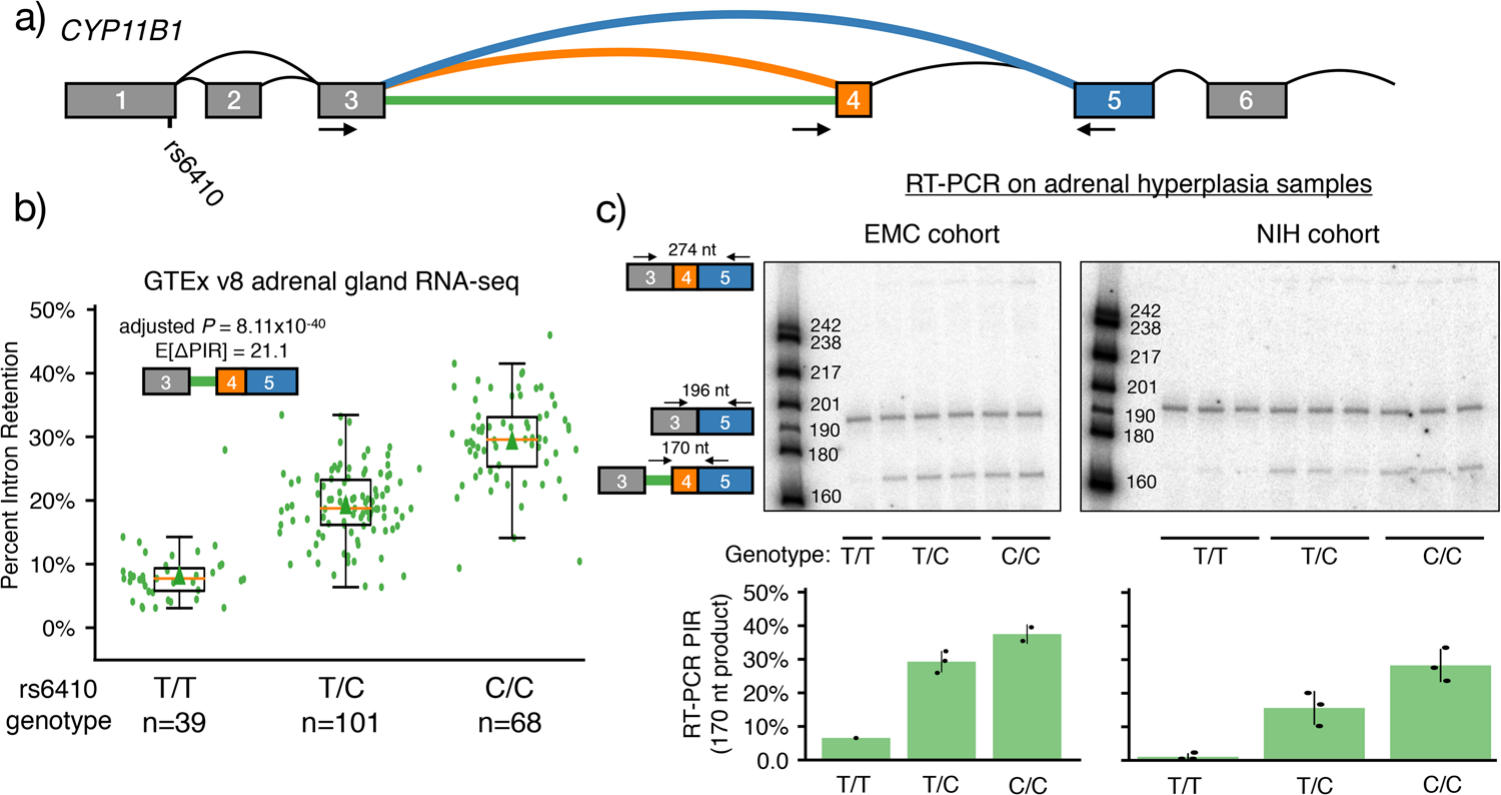
Association between rs6410 genotype and intron 3 retention. **a** Splice graph highlighting a local splicing variant (LSV) from exon 3. Blue junction represents skipping of exon 4; orange junction represents inclusion of exon 4; the green line represents retention of intron 3. Location of candidate SNP rs6410 is indicated and arrows indicate approximate locations for primers used for RT-PCR. Primer sequences are provided in Supplementary Data 8. **b** Scatterbox plot of MAJIQ quantifications for Percent Intron Retention (PIR) for intron 3 (green in the splice graph, cartoon in the plot inset), stratified by rs6410 genotype in GTEx. Boxes extend from the upper to lower quartile values of PIR per genotype and whiskers extend 1.5 times the interquartile range above the upper quartile and below the lower quartile. The line in the boxplot represents the group’s median value and the triangle represents the arithmatic mean. Legend indicates the Bonferonni adjusted *P* value for genotype effect and the expected change in PIR levels between homozygous C and homozygous T individuals. Source data for GTEx PIR values are available in Supplementary Data 9. **c** RT-PCR validation for the association between rs6410 genotype with intron 3 retention using six samples of adrenal hyperplasia from Erasmus MC (left) and nine samples from NIH (right). Primer locations and the expected products with sizes are shown on the left of the gels. Radiolabeled DNA ladder with known fragment sizes are included in the first lane of both gels. The full, uncropped gel images are available in Supplementary Fig. 8. Barplots below the gels indicate average PIR by genotype in each cohort with error bars indicating the sample standard deviation (where applicable) and show a significant association between rs6410 genotype and intron 3 retention in the Erasmus MC (Pearson *r*^2^ = 0.85, *P* = 0.009) and NIH (Pearson *r*^2^ = 0.91, *P* = 5.48 × 10^−5^) cohorts. Each samples’ PIR quantifcation is also included as a black dot on the barplot. Source data are available in Supplementary Data 10.

The sQTL pipeline identified a total of 292 significant associations (*P*_adjusted_ < 0.05), 166 of which had a meaningful difference in PSI between homozygous reference and homozygous alternative individuals (|ΔPSI| > 10%). All these associations involved two distinct splicing events, and many of the associated SNPs were in strong LD with rs6410 (Supplementary Data 5). The first LSV involved the retention of intron 3 (Fig. 2a, green), downstream of the exon 3 (Supplementary Fig. 5a), and upstream of an alternative cassette exon not included in the canonical protein isoform (exon 4, Fig. 2a, orange and Supplementary Figs. 5a, 6a for exon mapping to Uniprot isoforms). For this event, rs6410 achieved a strong association with alternative splicing outcomes, where the presence of the C allele was associated with increased intron 3 retention (ΔPSI = 21.1% versus homozygous T, *P* = 8.11 × 10^−40^, Fig. 2a, b, green), increased exon 4 inclusion (ΔPSI = 5.3% versus homozygous T, *P* = 4.29 × 10^−34^, Supplementary Fig. 5b, orange), and decreased splicing of exon 3 to exon 5 (ΔPSI = −26.4% versus homozygous T, *P* = 7.85 × 10^−43^, Supplementary Fig. 5b, blue). Notably, the most significant association was observed for rs4609179 (|ΔΨ| = −26.7%, *P* = 2.11 × 10^−46^ with the exon 4 skipping junction), a signal that also reached GWS in our meta-analysis (*P*_meta-analysis_ = 2.6 × 10^−11^; *r*^2^_with rs6471570_ = 0.85). We also inspected the raw RNA-seq alignments and found that these data clearly supported the presence of this sQTL with increased reads throughout intron 3 and exon 4 associated with the C allele of rs6410 (Supplementary Fig. 7a).

The second LSV with strong sQTL associations involved an alternative 3’ splice site in exon 9 of the canonical transcript isoform of *CYP11B1* (shorter, canonical 79 nt exon at chr8:142875234−142875312 vs. longer 148 nt exon at chr8:142875234−142875381) (Supplementary Figs. 5c, 6a, 7b). Our GWAS signal, rs6410, achieved *P* = 2.19 × 10^−27^ where the presence of the C allele was associated with decreased use of the intron-proximal 3ʹss which creates a longer 148 nt exon 9 that is not present in the canonical *CYP11B1* transcript or protein annotations (ΔPSI = −20.3% versus homozygous T, Supplementary Fig. 5c, red, Supplementary Fig. 6a). Notably, the annotated transcript containing the alternative junction had also its transcription start site at the 5’ end of exon 8 (chr8:142875712−142875878). One of the most significant associations in the GTEx samples involving this splicing event was with rs7016594 (*P*_meta-analysis_ = 6.4 × 10^−10^, *r*^2^_with rs6471570_ = 0,98; |ΔΨ| = −23.5%, *P* = 1.22 × 10^−44^ with the shorter exon). Additional splicing events had significant associations with various SNP genotypes (*P*_adjusted_ < 0.05), including rs6410 with increased cassette exon 10 inclusion (*P* = 3.39 × 10^−7^). However, given that the change in exon inclusion between homozygous individuals was small (ΔPSI = 7.7% with increased exon 10 inclusion with the C allele; Supplementary Fig. 5d, red), we did not focus further on these events.

In contrast to the GWAS results, the sex-stratified sQTL analyses did not reveal significantly stronger association of the rs6410 C allele with intron 3 retention in males (*β* = 0.78; *P* = 9.7 × 10^−24^; *N* = 122 samples) as compared to females (*β* = 0.65; *P* = 5.59 × 10^−14^; *N* = 86 samples) (*P*_het_ = 0.13). Detailed results of the sex-stratified sQTL analyses are shown in Supplementary Data 6 and 7.

### Splicing analysis replication in clinical samples

To validate our findings, we next performed reverse transcription-polymerase chain reaction (RT-PCR) experiments to quantify intron 3 retention and exon 4 inclusion using RNA from adrenal gland tissue from six donors obtained from patients of the Erasmus Medical Center (EMC) (see “Methods”). In the absence of healthy donors, we used the tissues from donors diagnosed with adrenal hyperplasia (see “Methods—RT-PCR”). In line with the MAF = 0.47 (T-allele), these six samples were distributed with one TT, three TC, and two CC genotypes at rs6410. Intron retention levels of intron 3 showed a clear correlation with genotype at rs6410 (Pearson’s *r*^2^ = 0.85, *P* = 0.009; Fig. 2c, left) recapitulating the observations made in the GTEx samples (Fig. 2b and Supplementary Fig. 8a).

We replicated the validation of the rs6410 sQTL using a second cohort of nine donors from an NIH repository diagnosed also with adrenal hyperplasia (no healthy donors were available) (see “Methods—RT-PCR”). rs6410 genotypes included three TT, three TC, and three CC individuals. The RT-PCR results from this cohort also showed a significant association between increased intron 3 retention and the C allele at rs6410 (Pearson’s *r*^2^ = 0.91, *P* = 5.48 × 10^−5^; Fig. 2c, right, Supplementary Fig. 8b).

Finally, we recalculated these correlations while excluding outlying samples. In the EMC samples, we excluded the single male, and in the NIH cohort, we excluded the single Hispanic individual. With these potential outliers excluded, we still observed very similar correlations between genotype and level of intron 3 retention that were consistent with the sQTL analysis from GTEx (EMC cohort Pearson’s *r*^2^ = 0.73, *P* = 0.064; NIH cohort Pearson’s *r*^2^ = 0.94, *P* = 7.68 × 10^−5^). We note that excluding the single male in the EMC cohort not only reduced the sample size to five individuals but also resulted in the exclusion of the single TT individual in this cohort.

Together these RT-PCR results from two independent cohorts of adrenal hyperplasia samples validate the sQTL detected by MAJIQ from GTEx samples.

### Variant effect prediction

Given that MAJIQ identified several variants as associated with changes in the *CYP11B1* splicing process, many of which were in LD with the candidate SNP (Supplementary Data 5), we further aimed to investigate a possible causal relationship between the other observed variants and splicing variations by employing splicing prediction tools. However, many tools, such as Spliceman2 ^17^, RegSNPs-Splicing^18^ and SPANR^19^, assume the splicing variations are cassette exons and are not appropriate for this specific case. Therefore, we assessed the effect on the strength of splice junctions using the MaxEntScan plugin in ENSEMBL’s Variant Effect Prediction (VEP) tool^20^. Overall, six SNPs that fell within annotated splice sites (final three bases of an exon and first six bases of an intron for 5ʹss; last 20 bases of an intron and first the three bases of an exon for 3ʹss) in *CYP11B1* were tested and included rs6395, rs7818826, rs61752786, rs4535, rs28418310 and rs5295) (Supplementary Methods). While this meant that we could not directly predict a variant effect for rs6410 since it did not fall within these splice site definitions, we hypothesized that other variants in LD with rs6410 across the relatively short *CYP11B1* locus (~6.6 kb) may be causal.

The strongest predicted effect was that of rs6395, an intronic variant located 10 bases upstream of the exon 9 alternative 3ʹ splice site event. The score difference of 1.236 indicates that the reference A allele (T on the sense strand) promotes stronger selection of the upstream 3ʹ splice site (longer exon 9) while the alternative C allele (G on the sense strand) favors the downstream 3ʹ splice site (shorter exon 9) (Supplementary Fig. 9a). This computationally predicted effect on splicing is supported by the MAJIQ sQTL pipeline which had this variant tied for the most significant association with this alternative 3ʹss event (*P* = 1.22 × 10^−44^) with almost no inclusion of the exon 9 extension in CC homozygotes (E[PSI] = 2.1%) and with higher inclusion of this extension in AA homozygotes (E[PSI] = 25.6%) (Supplementary Data 5 and Supplementary Fig. 9a). We also note that this SNP showed a high degree of LD with rs6410 (*r*^2^_with rs6410_ = 0.83), suggesting the observed association of rs6410 status with exon 9 alternative 3ʹss selection (Supplementary Fig. 5c) may be casually influenced through its LD with rs6395 variant.

The second strongest effect was predicted for rs7818826, a synonymous variant at the 5ʹ end of exon 4. This variant affects the known 3ʹ splice site consensus sequence and underscores the importance of the G nucleotide at the start of the exon. In line with this, the score difference of −0.636 indicates that the reference T allele (A on the sense strand) associates with greater skipping of exon 4 whereas the alternative C allele (G on the sense strand) corresponds with greater inclusion of exon 4 (Supplementary Fig. 9b). Here too, MAJIQ PSI quantifications were in line with the computational prediction, pointing to increased inclusion of exon 4 with a 5.4% median inclusion in homozygous CC individuals compared with no inclusion in TT carriers (PSI = 0.1%, Supplementary Data 5 and Supplementary Fig. 9b, orange). However, we note that the C allele was also strongly associated with increased intron 3 retention (Supplementary Fig. 9b, green). The remaining four variants did not reach a permissive MaxEntScan score change threshold of ±0.5, indicating no significant effect on splicing and their LD to rs6410 was either low or could not be determined from the 1000G panel (Supplementary Methods).

Flowchart for all previously described analyses is presented in Supplementary Fig. 10.

### Colocalization

Lastly, we have performed colocalization analysis between GWAS and sQTL (intron 3 retention) signals, obtaining high posterior probability (PP) 4 (96.7%) and low posterior probabilities PP0-PP3 (PP0 = 1.4 × 10^−74^, PP1 = 2.6 × 10^−9^, PP2 = 1.8 × 10^−67^, PP3 = 3.3 × 10^−2^). This provides additional level of evidence that GWAS of SA and this splicing event may share the same causal SNP.

## Discussion

We identify variants in *CYP11B1*, a gene encoding 11-β-hydroxylase (P-450_11β_), robustly associated with skeletal age of children from two independent multiethnic cohorts (*N* = 4557), with evidence of sex-heterogeneity (*P*_het_ = 0.02; *β*_girls_ = 0.10; *P* = 0.001; *β*_boys_ = 0.20; *P* = 1.7 × 10^−10^). Further, using MAJIQ sQTL in GTEx adrenal tissue samples (*N* = 208) and RT-PCR quantification in two clinical sets of adrenal tissue samples from patients with adrenal hyperplasia (*N* = 15), we demonstrate alternative splicing of *CYP11B1* as a plausible genetic mechanism of variants underlying the GWAS association.

The sex-stratified GWAS analyses showed a stronger effect of the variants at the *CYP11B1* locus in boys as compared to girls. Such effect attenuation in girls can be explained by their earlier age of sexual maturation, where the effect of adrenal activity on skeletal maturation is expected to be replaced by a gradual onset of estrogen production at this age^21,22^. Alternatively, girls aged 8.0−11.5 years old (similar to the age range of our study populations) are likely to have higher circulating levels for the majority of androgens, when compared to age-matched boys^23^, decreasing the impact of *CYP11B1* conversion. Altogether, the sex specificity of the GWAS associations was not observed in the sex-stratified sQTLs analyses, most likely due to the low statistical power for the latter analyses.

We observed that the rs6410 CC genotype is associated with increased retention of intron 3 and a slight increase in exon 4 inclusion with a decrease in exon 3 to exon 5 splicing (Fig. 2 and Supplementary Fig. 5b). There are several potential functional consequences for the splicing changes associated with the rs6410 CC genotype since both intron 3 retention and exon 4 inclusion are not utilized in the canonical version of this protein (Supplementary Fig. 6a, Uniprot ID P15538). Since intron 3 retention leads to a premature termination codon (PTC) just downstream of the unspliced 5ʹss of exon 3, these transcripts may be targets of nonsense-mediated decay (NMD) which may alter the level of gene expression^24^. However, because transcripts (including intron 3) were readily detectable by RT-PCR without the inhibition of NMD (Fig. 2c), it is possible that intron-retained mRNAs are detained in the nucleus or produce a truncated protein product corresponding to the first 132 amino acids of *CYP11B1*, as these downstream consequences of intron retention have been observed previously for other genes^25^. Increased exon 4 inclusion also has potential consequences for protein function. Alternative splicing often introduces disordered regions containing short linear peptide motifs^26^. Consistent with this, we found exon 4 introduced a disordered region (as predicted by IUPred2A^27^) into the otherwise highly structured canonical *CYP11B1* amino acid sequence (Supplementary Fig. 6b). Given that exon 4 region is unstructured, we also found a number of putative Short Linear Motifs (SLiMs) using the Eukaryotic Linear Motif tool^28^ (Supplementary Fig. 6c). This suggests post-translational modifications or protein−protein interaction sites may be added upon exon 4 inclusion (Supplementary Fig. 6c). Additionally, the inclusion of alternative exons may alter the conformation of *CYP11B1*, and thus its enzymatic activity. For example, a recent X-ray crystallography study of *CYP11B1* bound to fadrozole found that the B/Bʹ loop, just upstream of the residues inserted by alternative exon 4 (Supplementary Fig. 6b), flanks a channel from the protein surface to the active site^29^. Thus, the insertion of the disordered region upon exon 4 inclusion (Supplementary Fig. 6c) may alter *CYP11B1* activity.

Furthermore, the CC genotype at rs6410 was also associated with the increased use of the short version of exon 9 that is present in the canonical protein isoform (Supplementary Figs. 5c, 6a). The end of exon 8 and start of exon 9 encodes the K helix motif (KETLR) which contains the conserved glutamate (E371) and arginine (R374) residues that are part of the ERR-triad. The ERR-triad forms a coordinated hydrogen bonding network that is present in all P-450 proteins^30^ and is thought to help stabilize both heme-binding and the core structure^31^. While the exon 9 extension does not alter these conserved residues, it does add 23 residues to the region before the final arginine (R427) of the ERR-triad^30^ and thus may alter this key structural element.

Given the above, and following the model of CAH caused by loss of function mutation in *CYP11B1* gene, we propose that altered function of P-450_11β_, or its lower production, leads to the enhanced production of 11-deoxycortisol and 11-deoxycorticosterone, and hence, results in differences in skeletal maturation of pre-pubertal children. Likewise, reduced cortisol levels are expected to increase secretion of adrenocorticotropic hormone (ACTH) and consequently result in increased levels of androgens^32^, known to cause accelerated development. While future work is necessary to confirm the predictions that described alternative splicing events alter enzyme activity, we believe this is a reasonable hypothesis. The structural study referenced above compared their structure of *CYP11B1* to a previous structure of *CYP11B2*, which shares 93% amino acid sequence identity. While both proteins shared all active site residues, slight structural differences between the two highly similar proteins are consistent with their distinct functions and enzymatic activities^29^. This suggests that splicing variations from the canonical amino acid sequence of *CYP11B1* could lead to the repositioning of secondary structure elements and thus alter function. However, assays assessing enzyme activity, which we could not currently perform due to scarcity of the adrenal tissue and lacking measurements of adrenal steroids in the blood, warrant further investigation.

Using MAJIQ we demonstrated a number of other variants in the locus associated with changes in the *CYP11B1* splicing process. Therefore, we cannot unequivocally pinpoint a rs6410 as the real causal SNP underlying the GWAS association. Because rs6410 lies within exon 1, which is not directly adjacent to any of the significant splicing associations described above (Supplementary Fig. 5a), we hypothesized SNPs in LD that are located more proximally to the regulated splice sites may be causal. Indeed, our VEP analysis postulated other functional implications for rs6395 and rs7818826 that could also explain our findings. For example, rs6395 which is associated with the alternative 3ʹ splice site event in exon 9, lies within the polypyrimidine tract, upstream of the affected 3ʹss; and the U → G transversion is likely to result in inhibition of U2AF binding to this site during spliceosome assembly. Further, rs7818826 is located at the first nucleotide of exon 4. The variant (that increases the predicted splice site strength (A → G)) is associated with both increased exon 4 inclusion, in addition to intron 3 retention (Supplementary Fig. 9b). Mechanistically, this seems consistent with a study that found that cryptic exons (e.g. exon 4) can serve as decoys to the spliceosomal machinery and lead to non-productive interactions with constitutive splice sites leading to intron retention^33^. Since rs6395 and rs7818826 are in high LD (correlated) with our candidate variant rs6410 (*r*^2^ = 0.83 and 0.76, respectively), we cannot exclude the possibility that those splicing events could still be related to variation in the SA phenotype. We note that the VEP was limited to splice site score changes, as the events of interest (i.e. intron retention and alternative 3ʹss) were not classical cassette exons, which are the focus of available prediction tools. Therefore, additional variants outside of the consensus splice site sequences likely contribute to the associations between genotype and splicing outcome. For example, much work has shown that factors that regulate chromatin, transcription complexes, and/or Pol II elongation rates can have downstream effects on alternative splicing patterns^34^. Thus, we cannot rule out that SNPs located distal to the regulated events studied here (e.g. rs6410 genotype status with intron 3 retention or exon 9 splicing) may influence splicing outcomes by altering transcriptional regulation or elongation rates.

Together with the available literature^12,35–37^, our study further suggests a prominent role of naturally occurring variation in alternative splicing for complex diseases/traits, implying that evaluating the impact of genomic variants on splicing may be an integral part of clinical variant prioritization.

The T allele of rs6410 presenting faster maturation in children has been nominally associated with taller stature (*P* = 0.02) in children of 10−12 years of age^38^ and with shorter adult stature in GIANT UK Biobank GWAS (*P* = 1.4 × 10^−14^)^39^. This confirms pleiotropic effects of *CYP11B1* and is in line with a potential shared mechanism linking faster skeletal maturation and pediatric bone growth, to shorter adult stature due to earlier closure of epiphyseal plates^39,40^. Furthermore, through the process of growth and development skeleton reaches its peak bone mass^1^. This bone acquisition seems to be even more significant than the bone loss in adulthood when determining fracture risk in the elderly population^41^. Therefore, despite the current lack of information about the association between SA and bone mineral density, understanding genetic architecture of SA may help to develop approaches to maximize bone gain during maturation and optimize bone health^42^.

The clinical relevance of the reported locus may extend beyond the regulation of skeletal phenotypes. Increased estrogens and androgens in the serum have been associated with pre- and post-menopausal breast cancer development^43–46^. Further, rs6410 and *CYP11B1* have been associated with vertebral cross-sectional area (CSA) in older men assessed by quantitative computer tomography (L1-L2 region)^47^; with homozygous TT men showing lower vertebral CSA as compared to CC homozygous (*P* = 0.0002). This finding may indicate possible links between the pace of skeletal maturation and bone health in later life. Further, in line with the phenotypic presentation of monogenic forms of *CYP11B1* deficiency in humans and murine models, recent GWAS have also found the T allele of rs6410 associated with hypertension (*P* = 1.8 × 10^−10^)^40,48^.

Our study has certain limitations. A caveat in our validation experiments is that all adrenal samples (from both NIH and EMC) originated from adult donors diagnosed with adrenal hyperplasia (see “Methods”), which could affect our findings and diminish its generalizability to a healthy pediatric population. Nevertheless, the findings from the GTEx data originating from healthy individuals are consistent with those from samples with diverse adrenal hyperplasia disorders, supporting that these are likely naturally occurring alternative splicing variations, not modified by adrenal disease processes. SA was assessed in the Generation R Study on hand iDXA scans and in the BMDCS on hand radiographs. Even if radiographs offer higher resolution than DXA scans, skeletal age calculated from hand images of both techniques are comparable^49^. Furthermore, the data were analyzed independently in each cohort with the calculated standardized adjusted residuals as the outcome. In such a setting, we would not expect that the difference in resolution between DXA scans and radiographs bias our results. Lastly, the Greulich and Pyle method may demonstrate precision issues when utilized in Asian boys and African girls. Nevertheless, this could likely bias assessment of individual chronological age, but not the summary-level data drawn in a well-powered setting^50^.

In conclusion, this GWAS meta-analysis establishes one robust locus associated with SA variation in children. We suggest that changes in the *CYP11B1* splicing process and consequent alterations in P-450_11β_ activity and glucocorticoid biosynthesis constituting a plausible biological pathway underlying our GWAS observations. Our findings provide new leads to monitor normal skeletal maturation and bone health in general.

## Methods

### Study population

*The Generation R Study* is a multiethnic population-based pregnancy cohort study from fetal life until adulthood, established in Rotterdam, the Netherlands, at the Erasmus University Medical Center. Mothers of children born between April 2002 and January 2006 were invited to participate since pregnancy. The Generation R Study was designed with the aim to identify early environmental and genetic determinants of growth, development, and health, as described previously^51^. The Medical Ethics Committee of the Erasmus Medical Centre (MEC-2012-165) in Rotterdam, the Netherlands approved the study. At the beginning of each phase, children and their parent(s) provided written informed consent. This study included 3510 children of chronological age 8.5−12 years and with SA and GWAS data available. Applying genetic definition of ancestry^3^, 87.4% of children were classified as European, 7.0% as African, 3.8% as Asian, and 1.8% as of mixed ancestry.

*The Bone Mineral Density in Childhood Study* is a multiethnic, longitudinal study^52^. Healthy children from all major ethnic groups were enrolled from July 2002 to November 2003 within the age range from 6 to 16 years. A subsequent wave of enrollment of children 5 and 19 years of age occurred in the fourth year. Data were collected from five regional clinical centers in the United States: Children’s Hospital of Los Angeles (Los Angeles, CA), Cincinnati Children’s Hospital Medical Center (Cincinnati, OH), Creighton University (Omaha, NE), Children’s Hospital of Philadelphia (CHOP) (Philadelphia, PA), and Columbia University (New York, NY). Children were evaluated annually for up to 6 years after the baseline visit. An ancillary study to the BMDCS enrolled a cross-sectional cohort, ages 5−18 years, of approximately 500 children from two of the five BMDCS sites following identical study procedures. Written informed consent was obtained from a parent, or participant if older than 18 years. 1047 children 8−13 years old were included in the current study. Applying genetic definition of ancestry^3^, 73.6% of children were classified as European, 19.6% as African, 4,6% as Asian, and 2.2% as of mixed ancestry.

### Skeletal age assessment

In the Generation R Study, DXA-scans (iDXA; General Electric, formerly Lunar Corp., Madison, WI, USA) of the left hand were obtained for all 5758 subjects. All scans were performed using the same densitometer and performed by trained investigators. A patient’s SA was assessed by comparing the maturity indicators on the patient’s DXA scan to the standardized reference atlas according to the Greulich and Pyle method^49,53^, blinded to the age and ancestry of participants. DXA scans were assessed using optimal zoom, which could be adjusted by the observer. In case a discrepancy in bone development was found between the carpals and other regions, a mid-point SA was assigned. One trained observer performed all Greulich-Pyle assessments.

In the BMDCS, hand-wrist radiographs were acquired, and SA was assessed using the Greulich and Pyle atlas. Radiographs were graded by a pediatric radiologist for the BMDCS initial cohort (*N* = 842) and by a pediatric endocrinologist for the ancillary study (*N* = 205). Both were blinded to the ancestry and age of the study participant and were instructed not to interpolate between bone age categories.

### Genotyping

In the Generation R Study, genotyping was performed using Illumina HumanHap 610 or 660 Quad chips (Illumina Inc., San Diego, USA). The detailed quality control process is described elsewhere^54^. Briefly, prior to imputation, the variant exclusion was made if the MAF was <1%, SNP call-rate <97.5%, and/or departure from Hardy−Weinberg equilibrium (HWE) proportions was observed (<*P* < 10^−7^); whereas sample exclusion was based on sex mismatch, and low genotype call (98%). Imputation to the 1000G Phase 3 (version 5) reference panel was carried out in the Michigan Imputation Server using the EAGLE2/minimac3 software^55^. In subsequent analyses, only SNPs of sufficient imputation quality (*r*^2^ = 0.30) and MAF < 1% were analyzed.

In the BMDCS, genotyping was performed using the Illumina Infinium II OMNI Express plus Exome BeadChip technology (Illumina, San Diego, CA, USA). Samples with sex discrepancy, sample replicates, siblings, and/or low genotype quality were excluded together with SNPs with MAF < 1% and SNP call rates < 95%. Imputation was performed using IMPUTE2 software and 1000 G Phase 3 (version 5) as the reference panel^55^. In subsequent analyses, only SNPs of sufficient imputation quality (*r*^2^ = 0.30) and MAF < 1% were analyzed.

### Heritability

Heritability of SA was estimated in the Generation R Study, in the subset of children classified as European applying genetic definition of ancestry^3^. To do so, the GCTA tool and restricted maximum likelihood (REML) approach were used^56^. The genetic relationship matrix was computed for the European subsample of children, defined as such using principal components as in detail described elsewhere^54^. To diminish the possibility of introducing bias, in the GCTA analysis, one child from every closely related pair (relatedness cut-off 0.025) was removed, retaining the maximum number of children in the dataset^57^. Eventually, this reduced the European subsample for heritability analysis to 1797 participants.

### FINEMAP

FINEMAP^9^ was used to create a credible set of SNPs containing the true causal SNP with a 95% of certainty using summary statistics and LD matrices for chromosome 8 (embedding GWS locus). LD blocks in chromosome 8 were defined using LDetect^58^ and LD-matrices were created with CAUSALdb^59^, using EUR population from 1000G reference panel (resembling the most our admixed, but predominantly European population). Lastly, maximum number of causal SNPs was set at 1, given no secondary associated SNPs were found within the GWS locus^60^.

### Gene expression

#### GTEx tissues

To assess the expression of the *CYP11B1* gene, raw gene read counts were downloaded from the GTEx v8 on 17 May 2020. Raw gene expression values for each specific tissue sample were normalized using the trimmed mean of M-values method in the *edgeR* package^61^. Expression values were then log_10_ transformed using counts per million function in order to prevent negative values. Genes that showed transformed expression values lower than 0 in more than half of all specific tissue samples were excluded from further analyses. Fifty-three paired DE analyses between the tissues were then performed in an iterative manner. The two processed tissue gene expression datasets were merged while keeping overlapping genes. DE analysis was then carried out between the two tissue gene expressions using the glm approach in the *edgeR* package.

#### Primary bone tissue

Expression of the *CYP11B1* gene was further evaluated in the RNA-Seq dataset generated from 71 iliac crest biopsies and 50 subchondral femoral head fragments obtained after hip replacement surgery. The donors were Norwegian women and men (age range 49−90 years). All the prepared libraries were analyzed in a single batch. TruSeq RNA Library prep kit V2 (Illumina, USA) was used to capture poly(A) RNA from 1000 ng total RNA. Subsequently, cDNA was prepared to which single indexed adapters were ligated. The material was then cloned for 13 cycles with PCR. Paired-end sequencing of 2 × 50 bp was performed using the Illumina HiSeq2000 (Illumina, USA) platform to obtain at least 6,000,000 reads per library. They were aligned to reference genotype (GRCh37.p13, gencode release 19) using STAR 2-pass methodology. Picard tools were used to add read groups to bam files, reorder contigs according to the reference file and remove PCR duplicates. In the next step, GATK was used to split cigar reads, realign reads around indels, and recalibrate quality scores of the resulting reads. Picard was again used to extract QC metrics. In the end, raw transcription level expression values were extracted using feature counts. Expression data were normalized in the same manner as already described before. Genes not expressed in at least 75% of libraries were excluded from the analysis.

### Variant prioritization

The best candidate variant for sQTL analysis was chosen based on its location in the gene, the GWAS *P* value (having LD_with_top_variant_ > 0.80), and the results of FINEMAP. Among GWS variants, the prioritized variant was the one closest to the first exon intron−exon boundary, second most significant in our dataset, and having the second highest log10 Bayes factor.

### Splicing QTL analysis

We considered the 208 paired-end RNA-seq experiments from GTEx v8 adrenal donors that were of sufficient quality (RIN > 6 (SMRIN) and not flagged for removal (SMTORMVE)) that had whole-genome sequencing data available. These samples were mapped to the hg38 genome annotation using STAR 2.5.3a with the option–alignSJoverhangMin 8. Sorted and indexed alignments were built into splice graphs by MAJIQ 2.1 build where LSVs and novel splice junctions were incorporated into the splice graph if they were supported by two-thirds of the samples with a minimum of three reads across two start positions per sample. The coverage threshold for intron retention detection was increased (–min-intronic-cov 10) to account for the high expression levels of *CYP11B1* in the adrenal gland. LSV junctions for each sample were then quantified using percentage of inclusion of the junctions; percent spliced in (PSI) in MAJIQ 2.1.

We queried 762 genomic variants within 10 kb of the *CYP11B1* locus as the sQTL search space. Genotypes of adrenal donors at these variants were determined by the vcf file from GTEx v8 based on SHAPEIT2 phased analysis of 838 individuals’ whole-genome sequencing. To limit the number of statistical tests performed, our pipeline imposes strict filters for the quantifiability of LSV-variant associations. In brief, tested variants must have a minimum minor allele count of 5 and minimum MAF of 0.1 among the sets of samples for which junction PSI was quantifiable. Additionally, we required the variant to occur within 10 kb of any exon associated with the splicing event. While we considered novel splice junctions in our detection of LSVs, we only quantified inclusion and performed sQTL analysis on annotated splice junctions since no de novo junctions were highly utilized in the final transcript in the adrenal samples (median PSI < 5% and max PSI < 8% for all de novo junctions across all samples, meaning no de novo junction can reach the |ΔPSI| > 10% threshold).

Given the above, the following sQTL pipeline was performed to test for significant associations. For all LSVs that passed the above thresholds, all junction PSI values were quantile-normalized to a standard Gaussian distribution. Next, various covariates, provided by GTEx v8, were regressed out of the transformed PSI values as potential confounding factors. These included known covariates (sex, library preparation, and sequencing platform), “hidden” covariates (15 PEER factors based on splicing quantifications), and the first five principal components based on donor genotype. For each junction in each LSV, the statistic of linear association between the transformed, normalized PSI value and the genotype residuals was calculated and significance was assessed based on the null hypothesis of a slope of zero. Finally, a Bonferroni multiple hypothesis test correction was applied based on the 553 total LSV to SNP pairs tested and we focused on the events. The code for this pipeline in addition to the arguments used to reproduce this analysis are provided in a repository at https://bitbucket.org/biociphers/majiq_sa_gwas_sqtl.

Because LSVs for certain events can be partially redundant^62^ (e.g. the source LSV with reference exon 9 and the target LSV with reference exon 11 both quantify cassette exon 10 inclusion), we only reported source LSV sQTL associations in our analysis. Additionally, while many associations were significant, we chose to focus on the LSV to SNP associations that had a substantial difference in median PSI values between the homozygous reference and homozygous alternative allele individuals of 10% or more (|ΔPSI| > 10%).

Sex-stratified sQTL analyses were performed following the same protocols.

### RT-PCR

Total RNA was purified from each of 15 donor adrenal glands, nine supplied by the NIH (8 Caucasian and 1 Hispanic girl; age range 3−11 years, all diagnosed with micro-nodular adrenal hyperplasia) and six provided by Erasmus MC (5 Caucasian women and 1 Caucasian man; age range 50−79 years, 4 diagnosed with adrenal hyperplasia due to ectopic adrenocorticotropic hormone (ACTH) secretion and 2 with ACTH independent macro-nodular hyperplasia) and approved by the medical ethical committees of both institutes.

Low cycle RT-PCR was performed using two 32P-labeled, sequence-specific forward primers (one for exon 3 and one for the 3ʹ end of intron 3) and a reverse primer for exon 5 (Supplementary Data 8)^63^. This two-forward primer strategy was used to more accurately quantify intron 3 inclusion since the reverse transcription step has a bias against longer products and amplifying through this ~1.2 kb intron would be much less efficient compared to the smaller spliced products (196 nt or 274 nt products).

A subset of samples was optimized for PCR cycle number to ensure the amplification of products was within the linear range. All samples were then run for 25 cycles. Products were run on a 2.5% acrylamide gel and were quantified by densitometry with the use of a Typhoon PhosphorImager (Amersham Biosciences, UK) with background correction. For each sample, the percent intron retention (PIR) was quantified as the average band intensity of the 170 nt product divided by the sum of all relevant products (the 170 nt intron retention band, the 196 nt exon 4 skipping product, and the 274 nt exon 4 inclusion product).

### Colocalization

Bayesian colocalization analysis was performed using “coloc” R package with default settings^64^. It was applied on overlapping SNPs (40 SNPs from the GWAS and sQTL analysis), taking intron 3 retention as a splicing event of interest. As suggested by the authors, colocalization was considered to be present when PP4 > 0.8 (80%).

### Statistics and reproducibility

Standardized residuals of SA corrected for age and BMI (plus ten genomic principal components in the Generation R Study or visit site and initial/ancillary cohort status in the BMDCS) were calculated separately for boys and girls in both cohorts and used as outcomes. Association between genotypes and SA was tested using linear regression in the Generation R Study and linear mixed models in BMDCS. Similarly, in the Generation R, standardized residuals of SA corrected for age and BMI were computed separately in boys and girls and used for the heritability analysis. Least square SA means corrected for age, sex, BMI, and ten genomic PCs were calculated (in the Generation R population only) to quantify differences in the pace of skeletal maturation across different rs6410 genotypes. Both sex-stratified and joint association analyses were performed. The fixed-effects inverse variance method implemented in METAL^65^ was used for the meta-analysis. GWS was set at *P* < 5 × 10^−8^. Differences in the results across sexes were evaluated using EasyStrata. GCTA conditional analysis was performed to identify secondary associated SNPs within GWS locus as described elsewhere in detail^66^ using the same significance threshold. In the sQTL analysis, a total of 553 LSV−SNP pairs were tested for significant association. For each LSV, missing genotypes were imputed from the set of PSI-quantifiable samples. PSI values were quantile-normalized to a standard normal distribution. We used covariates provided by GTEx v8 as potential confounding factors including the subject’s sex, genotype PCs, 15 “hidden” confounding PEER matrix factors based on splicing quantifications across individuals, sequencing platform, and sequencing protocol. These confounding factors were regressed out from both the transformed PSI and the imputed genotypes. Finally, we computed the statistic of linear association between the PSI and genotype residuals against the null hypothesis of 0 slope. Association *P* values were adjusted using a conservative Bonferroni correction.

### Reporting summary

Further information on research design is available in the Nature Research Reporting Summary linked to this article.

## Supplementary information

Supplementary Material

Description of Supplementary Files

Supplementary Data 1

Supplementary Data 2

Supplementary Data 3

Supplementary Data 4

Supplementary Data 5

Supplementary Data 6

Supplementary Data 7

Supplementary Data 8

Supplementary Data 9

Supplementary Data 10

Supplementary Data 11

Reporting Summary

## Data Availability

GWAS summary statistics are publicly available at the GEFOS website (https://www.gefos.org/). The RNA-Seq data of the primary bone tissue are publicly available at the GEFOS website (https://www.gefos.org/), and the SRA (accession number: PRJNA764826). RNA-Seq data from adrenal tissue can be downloaded from the GTEx portal (https://gtexportal.org/home/datasets). Source data for the Fig. 1 is available in Supplementary Data 5 (sQTL locus zoom; http://locuszoom.org) or publicly available at the GEFOS website (https://www.gefos.org/) (GWAS locus zoom; http://locuszoom.org). Source data for Fig. 2 is available in Supplementary Data 9 and 10. Amino acid sequences for isoforms of CYP11B1 considered in this study are available in Supplementary Data 11.

## Code availability

The code for the pipeline and the arguments used to reproduce sQTL analysis are provided in a repository at https://bitbucket.org/biociphers/majiq_sa_gwas_sqtl. Other codes are available on reasonable request.
