# Supplementary material for: CYP11B1 variants influence skeletal maturation via alternative splicing
Source: Commun Biol. 2021 Nov 9;4:1274. doi: 10.1038/s42003-021-02774-y (PMC8578655; doi:10.1038/s42003-021-02774-y)
Supplement: Supplementary file 2 — Description of Supplementary Files [file 42003_2021_2774_MOESM2_ESM.pdf]

Description of Additional Supplementary Files

File name: Supplementary Data 1

Description: Variants associated with SA at GWS level.

File name: Supplementary Data 2

Description: Credible set of SNPs created using FINEMAP.

File name: Supplementary Data 3

Description: Variants associated with SA at suggestive level obtained from combined meta-analysis.

File name: Supplementary Data 4

Description: Variants associated with SA at GWS level in boys obtained from sex stratified meta-analysis.

File name: Supplementary Data 5

Description: sQTL analysis for CYP11B1 based on all 208 GTEx adrenal donors.

File name: Supplementary Data 6

Description: Sex stratified sQTL analysis within males.

File name: Supplementary Data 7

Description: Sex stratified analysis within females.

File name: Supplementary Data 8

Description: Description of RT-PCR primers.

File name: Supplementary Data 9

Description: Percent intron retention (PIR) values for CYP11B1 intron 3 for 208 GTEx adrenal donors based on rs6410 genotype.

File name: Supplementary Data 10

Description: Percent intron retention (PIR) values for CYP11B1 intron 3 for RT-PCR validations from adrenal hyperplasia samples.

File name: Supplementary Data 11

Description: Amino acid sequences for potential CYP11B1 isoforms.
