# Supplementary material for: CYP11B1 variants influence skeletal maturation via alternative splicing
Source: Commun Biol. 2021 Nov 9;4:1274. doi: 10.1038/s42003-021-02774-y (PMC8578655; doi:10.1038/s42003-021-02774-y)
Supplement: Supplementary file 14 — Reporting Summary [file 42003_2021_2774_MOESM14_ESM.pdf]

nature research

Corresponding author(s): Prof. Dr. Fernando Rivadeneira

Last updated by author(s): 01.05.2021

Reporting Summary

Nature Research wishes to improve the reproducibility of the work that we publish. This form provides structure for consistency and transparency in reporting. For further information on Nature Research policies, see our Editorial Policies and the Editorial Policy Checklist.

Statistics

For all statistical analyses, confirm that the following items are present in the figure legend, table legend, main text, or Methods section.

n/a	Confirmed	
☐	☒	The exact sample size (*n*) for each experimental group/condition, given as a discrete number and unit of measurement
☐	☒	A statement on whether measurements were taken from distinct samples or whether the same sample was measured repeatedly
☐	☒	The statistical test(s) used AND whether they are one- or two-sided<br>*Only common tests should be described solely by name; describe more complex techniques in the Methods section.*
☐	☒	A description of all covariates tested
☐	☒	A description of any assumptions or corrections, such as tests of normality and adjustment for multiple comparisons
☐	☒	A full description of the statistical parameters including central tendency (e.g. means) or other basic estimates (e.g. regression coefficient) AND variation (e.g. standard deviation) or associated estimates of uncertainty (e.g. confidence intervals)
☐	☒	For null hypothesis testing, the test statistic (e.g. F, t, r) with confidence intervals, effect sizes, degrees of freedom and P value noted<br>*Give P values as exact values whenever suitable.*
☒	☐	For Bayesian analysis, information on the choice of priors and Markov chain Monte Carlo settings
☒	☐	For hierarchical and complex designs, identification of the appropriate level for tests and full reporting of outcomes
☐	☒	Estimates of effect sizes (e.g. Cohen's *d*, Pearson's *r*), indicating how they were calculated

Our web collection on statistics for biologists contains articles on many of the points above.

Software and code

Policy information about availability of computer code

Data collection	Data includes phenotypic, genotypic and RNA-seq information. Method section contains detailed description on how these were obtained in each cohort.
Data analysis	Data analysis is in detail described in the method section together with all softwares and tools used (such as MAJIQ v.2.1, MaxEntScan/ ENSEMBL's Variant Effect Prediction tool, METAL, GCTA, different R packages as described throughout the methods).

For manuscripts utilizing custom algorithms or software that are central to the research but not yet described in published literature, software must be made available to editors and reviewers. We strongly encourage code deposition in a community repository (e.g. GitHub). See the Nature Research guidelines for submitting code & software for further information.

Data

Policy information about availability of data

All manuscripts must include a data availability statement. This statement should provide the following information, where applicable:

- Accession codes, unique identifiers, or web links for publicly available datasets
- A list of figures that have associated raw data
- A description of any restrictions on data availability

"GWAS summary results will be made available through the GEFOS Consortium website (www.gefos.org). Scripts for the MAGIQ analysis will be made available at https://bitbucket.org/biociphers/majiq_sa_gwas_sqtl

Field-specific reporting

Please select the one below that is the best fit for your research. If you are not sure, read the appropriate sections before making your selection.

☒ Life sciences ☐ Behavioural & social sciences ☐ Ecological, evolutionary & environmental sciences

For a reference copy of the document with all sections, see nature.com/documents/nr-reporting-summary-flat.pdf

Life sciences study design

All studies must disclose on these points even when the disclosure is negative.

Sample size	We have performed this study in 4,557 healthy children from two cohorts having complete phenotypic and genotypic information. In the context of GWAS, this is a relatively small sample size, which still allowed us to detect one locus strongly associated with skeletal maturation. However, we are aware that increase in sample size in future may lead to the discovery of novel loci associated with skeletal maturation. For splicing analysis we included all (208) adrenal gland RNA-seq samples from GTEx that passed quality control and with whole genome sequencing data available. For the RT-PCR, we extracted adrenal gland RNA from each of 15 available donors.
Data exclusions	Given that the two cohorts comprise population of healthy children, the only exclusions were based on absence of phenotypic and genotypic data availability (including QC failure). Also, adrenal RNA-seq samples not passing quality control (see Method section) were excluded.
Replication	BMDCS was used to perform replication after the discovery GWAS was performed in the Generation R only. Also, splicing findings obtained using MAJIQ software were further replicated using RT-PCR in adrenal samples from two sets of samples comprising 15 independent donors.
Randomization	Alleles are randomized at the conception, so when looking into splicing, our samples were randomized based on their genotypes for the SNP (s) of interest.
Blinding	When data was initially collected, we were blinded for the genotypic information of donors which we used later on to classify our samples.

Reporting for specific materials, systems and methods

We require information from authors about some types of materials, experimental systems and methods used in many studies. Here, indicate whether each material, system or method listed is relevant to your study. If you are not sure if a list item applies to your research, read the appropriate section before selecting a response.

Materials & experimental systems

n/a	Involved in the study	
☒	☐	Antibodies
☒	☐	Eukaryotic cell lines
☒	☐	Palaeontology and archaeology
☒	☐	Animals and other organisms
☐	☒	Human research participants
☐	☒	Clinical data
☒	☐	Dual use research of concern

Methods

n/a	Involved in the study	
☒	☐	ChIP-seq
☒	☐	Flow cytometry
☒	☐	MRI-based neuroimaging

Human research participants

Policy information about studies involving human research participants

Population characteristics	Total RNA from adrenal gland was purified for each of the 15 donors; 9 supplied by the NIH (8 Caucasian and 1 Hispanic girl; age range 3-11 years, all diagnosed with micro-nodular adrenal hyperplasia) and 6 provided by Erasmus MC (5 Caucasian women and 1 Caucasian man; age range 50-79 years, 4 diagnosed with adrenal hyperplasia due to ectopic adrenocorticotropic hormone [ACTH] secretion and 2 with ACTH independent macro-nodular hyperplasia)
Recruitment	Due to the lack of healthy donors, we utilized RNA data from donors which were available in the two institutes. They are all diagnosed with adrenal hyperplasia and their age range differ from the age range of children included in our GWAS. This requires caution when generalizing these findings on healthy pediatric population.
Ethics oversight	The utilization of adrenal gland RNA data was approved by the medical ethical committees of both NIH and ErasmusMC.

Note that full information on the approval of the study protocol must also be provided in the manuscript.

Clinical data

Policy information about clinical studies

All manuscripts should comply with the ICMJEguidelines for publication of clinical research and a completedCONSORT checklist must be included with all submissions.

Clinical trial registration	NA
Study protocol	NA
Data collection	NA
Outcomes	NA
