# Supplementary material for: CYP11B1 variants influence skeletal maturation via alternative splicing
Source: Commun Biol. 2021 Nov 9;4:1274. doi: 10.1038/s42003-021-02774-y (PMC8578655; doi:10.1038/s42003-021-02774-y)
Supplement: Supplementary file 1 — Supplementary Material [file 42003_2021_2774_MOESM1_ESM.docx]

***CYP11B1* Variants Influence Skeletal Maturation Via Alternative Splicing**

Olja Grgic^1,2,3†^, Matthew R. Gazzara^4,5†^, Alessandra Chesi^6†^, Carolina Medina-Gomez^1,3,7^, Diana L. Cousminer^4,8^, Jonathan A. Mitchell^9,10^, Vid Prijatelj^1,2,3^, Jard de Vries^1^, Enisa Shevroja^1,11^, Shana E. McCormack^9,12^, Heidi J. Kalkwarf^13^, Joan M. Lappe^14^, Vicente Gilsanz^15,16^, Sharon E. Oberfield^17^, John A. Shepherd^18^, Andrea Kelly^9,12^, Soroosh Mahboubi^19^, Fabio R. Faucz^20^, Richard A. Feelders^1^, Frank H. de Jong^1^, Andre G. Uitterlinden^1,3,7^, Jenny A. Visser^1^, Louis R. Ghanem^9,10^, Eppo B. Wolvius^2,3^, Leo J. Hofland^1^, Constantine A. Stratakis^20^, Babette S. Zemel^9,10^, Yoseph Barash^4‡^, Struan F.A. Grant^4,6,8,9,12‡^, Fernando Rivadeneira^1,3,7*‡^

*^1^Erasmus MC, University Medical Center Rotterdam, Dr Molewaterplein 40, 3015 GD, The Netherlands, Department of Internal Medicine;*

*^2^Erasmus MC, University Medical Center Rotterdam, Dr Molewaterplein 40, 3015 GD, The Netherlands, Department of Oral and Maxillofacial Surgery;*

*^3^Erasmus MC, University Medical Center Rotterdam, Dr Molewaterplein 40, 3015 GD, The Netherlands, The Generation R Study;*

*^4^Perelman School of Medicine, University of Pennsylvania, 2615 Civic Center Boulevard, Philadelphia, PA 19104, USA, Department of Genetics;*

*^5^Perelman School of Medicine, University of Pennsylvania, 2615 Civic Center Boulevard, Philadelphia, PA 19104, USA, Department of Biochemistry and Biophysics;*

*^6^Children’s Hospital of Philadelphia, 3401 Civic Center Boulevard, Philadelphia, PA 19104, USA, Center for Spatial and Functional Genomics;*

*^7^Erasmus MC, University Medical Center Rotterdam, Dr Molewaterplein 40, 3015 GD, The Netherlands, Department of Epidemiology;*

*^8^Children's Hospital of Philadelphia, 3401 Civic Center Boulevard, Philadelphia, PA 19104, USA, Division of Human Genetics;*

*^9^Perelman School of Medicine, University of Pennsylvania, 3400 Civic Center Boulevard, Philadelphia, PA 19104, USA, Department of Pediatrics;*

*^10^The Children's Hospital of Philadelphia, 3401 Civic Center Boulevard Philadelphia, PA 19104, USA,* [*Division of Gastroenterology, Hepatology, and Nutrition*](https://www.chop.edu/centers-programs/division-gastroenterology-hepatology-and-nutrition)*;*

*^11^Center of Bone Diseases, Lausanne University Hospital, Rue du Bugnon 46, Lausanne 1011, Switzerland, Bone and Joint Department;*

*^12^Children's Hospital of Philadelphia, 3401 Civic Center Boulevard Philadelphia, PA 19104, USA, Division of Endocrinology and Diabetes;*

*^13^Cincinnati Children's Hospital Medical Center, University of Cincinnati College of Medicine, 3333 Burnet Ave, Cincinnati, OH 45229, USA, Department of Pediatrics;*

*^14^Creighton University, 2500 California Plaza, Omaha, NE 68178, USA, Division of Endocrinology;*

*^15^Children's Hospital Los Angeles, Keck School of Medicine, University of Southern California, 1975 Zonal Ave, Los Angeles, CA 90033, USA, Division of Orthopedic Surgery;*

*^16^Children's Hospital Los Angeles, Keck School of Medicine, University of Southern California, 1975 Zonal Ave, Los Angeles, CA 90033, USA, Department of Radiology;*

*^17^Morgan Stanley Children’s Hospital, Columbia University Irving Medical Center, 622 West 168th Street, PH17 W 307, New York, NY 10032, USA, Division of Pediatric Endocrinology;*

*^18^University of Hawai’i Cancer Center, 701 Ilalo St, Honolulu, HI 96813, USA, Cancer Epidemiology;*

*^19^Children’s Hospital of Philadelphia, 3401 Civic Center Boulevard, Philadelphia, PA 19104, USA, Department of Radiology;*

*^20^Eunice Kennedy Shriver National Institute of Child Health and Human Development (NICHD), National Institutes of Health, 6710 Rockledge Dr. Bethesda, MD 20817, USA, Section on Endocrinology and Genetics;*

Competing interests: The authors declare no competing interests.

^†^ These authors contributed equally to this work.

^‡^ These authors jointly supervised this work.

^*^ Fernando Rivadeneira,

Department of Internal Medicine

Erasmus University Medical Center Rotterdam

Postbus 2040, Rotterdam, 3000 CA

The Netherlands

e-mail: f.rivadeneira@erasmusmc.nl

**SUPPLEMENTARY MATERIAL**

**Supplementary Figure 1.** **Manhattan plot of association statistics obtained from the combined meta-analysis**. Each dot represents one SNP, x-axis is its chromosomal position- build 37 NCBI, on the y-axis the –log10 (*P* value) is reported; horizontal red and blue lines mark the GWS threshold (*P*<5x10^-8^) and suggestive threshold (*P*<1x10^-5^), respectively. Top variant together with its minor allele frequency (MAF) are given in a red square. Figure also includes Q-Q plot obtained from the joint meta-analysis; plot shows the distribution of expected against observed *P* values, diagonal red line represents the null distribution, each dot represents one SNP; λ – genomic inflation fac

**Supplementary Figure 2.** **Miami plot of association statistics obtained from sex stratified meta-analisis**; each dot represents one SNP, x-axis is its chromosomal position- build 37 NCBI, on the y-axis the –log10 (*P* value) is reported separately for boys and girls; horizontal red lines mark the GWS threshold (P<5x10^-8^). Figure also includes Q-Q plot (in red for girls and in blue for boys) obtained from the sex stratified meta-analysis; plot shows the distribution of expected against observed p-values; diagonal red line represents the null distribution, each dot represents one SNP; λ – genomic inflation factor

**Supplementary Figure 3.** **Expression of *CYP11B1* across different tissues in the GTEx project (v8).** x-axis: 54 tissues available, y-axis: expression values presented in transcripts per milion (TPM); expression scale is log transformed (with 1 being added to prevent negative values). Boxplots demonstrate medians with 25^th^ ans 75^th^ percentiles, grey points- outliers (above/below 1.5x the interquartile range)

**Supplementary Figure 4. Relative skeletal age (skeletal age - chronological age) across different rs6410 genotypes (x-axis) (The Generation R Study; N=3,510);** Boxplot legend: black bar – median value, lower/upper margin of boxplot – 25th/75th percentile, T bars – the minimum and maximum value, circle – outlier; Difference in skeletal age among the rs6410 genotype groups was assessed using least square means corrected for age, sex, BMI and 10 genomic PCs obtaining *P*<0.0001.

**Supplementary Figure 5: LSV splice graphs with SNP details and PSI by rs6410 genotypes. a)** Full gene splice graph for *CYP11B1* detected from adrenal gland tissues with approximate location of candidate SNP rs6410. Numbered gray boxes represent exons, black arcs represent split RNA-seq reads corresponding to splice junctions, and thin gray rectangles represent intron retention events detected by MAJIQ. The terminal exon 11 is truncated for space. **b)** (top) LSV depicted in Figure 2 showing splicing patterns around cassette exon 4 with approximate SNP locations. (bottom) scatterbox plots showing Percent Selected Index (PSI) or Percent Intron Retention (PIR) for all samples stratified by genotype at rs6410 for each splicing outcome from the LSV: green, intron 3 retention; orange, exon 4 inclusion; and blue, exon 4 skipping. Plot insets depict the predicted isoform associated with each junction and show significance of association between PSI and rs6410 status (with Bonferroni correction) as well as the expected difference in splicing between homozygous C and homozygous T individuals (E[ΔPSI]). **c)** Same as b) but for the Exon 9 alternative 3’ss event leading to a long exon 9 (red) or the canonical short exon 9 (blue). **d)** Same as b) but for the cassette exon 8 skipping (blue) or inclusion (red) which had a significant association with rs6410 genotype, but lacked a large E[ΔPSI] between homozygous individuals. For all scatter boxplots, boxes extend from the upper to lower quartile values of PSI per genotype group and whiskers extend 1.5 times the interquartile range above the upper quartile and below the lower quartile. The line in the boxplot represents the group’s median value and the triangle represents the arithmatic mean.

**Supplementary Figure 6. Transcript and protein isoform annotations of *CYP11B1* and putative functional alterations upon alternative splicing**. **a)** (top) Full gene splice graph for *CYP11B1* detected from adrenal gland tissues. Numbered gray boxes represent exons, black arcs represent split RNA-seq reads corresponding to splice junctions, and thin gray rectangles represent intron retention events detected by MAJIQ. The terminal exon 11 is truncated for space. (bottom) Splice isoforms that correspond to full protein isoform and protein fragment annotations provided by Uniprot for *CYP11B1*. The canonical protein isoform (P15538) is labeled with an asterisk. See Supplementary Data 11 for full amino acid sequences **b)** (top) IUPred2A graphs (**Mészáros et al. 2018**) that predict regions of protein disorder (IUPred2, red) and probability of regions containing a disordered binding region (Anchor2, blue) for the canonical protein isoform (P15538) with approximate exon to residue position mapping under the graph. Also included are approximate locations of secondary structure motifs for *CYP11B1* from Uniprot based on the crystal structure of this canonical isoform with regions corresponding to the active site channel (**Brixius-Anderko & Scott, 2019**) and the highly conserved E-R-R triad in all CYP proteins (**Prosser et al. 2006**) that overlap regions of alternative splicing. (bottom) IUPred2A graph, as above, for the protein isoform including exon 4 (P15538+exon4). **c)** Amino acid sequence of exon 4 that is added to the canonical protein isoform and information related to putative Short Linear Motifs (SLiMs) that are added to the protein upon exon 4 inclusion as predicted by the Eukaryotic Linear Motif (ELM) tool (**Kumar et al. 2020**). The final column shows the total number of putative motifs located elsewhere in the canonical protein.

**Supplementary Figure 7. RNA-seq raw coverage of significant alternative splicing events. a)** Raw read pileups spanning exons 1, 2, 3, 4, and 5. Three GTEx samples are presented here whose genotypes at rs6410 are, from top to bottom, T/T, T/C, and C/C. RefSeq transcripts are displayed below these for reference. The blue highlighted portion spans the exon 3-4 junction and highlights the trend of increased retention of intron 3 levels across genotype. Coverage of the intron between exons 3 and 4 is high, particularly in comparison with coverage of the intron between exons 8 and 9 in b. **b)** Raw read pileups spanning exons 8 and 9. Each track above is a representative donor of each genotype at rs6410 in GTEx. Red boxes are drawn around the alternative and constitutive portions of exon 9 to highlight the trend in 3’ splice site usage levels over genotype.

**Supplementary Figure 8: Uncropped RT-PCR gel images** **a)** Full uncropped gel image for RT-PCR of EMC cohort samples after overnight exposure used for Figure 2. The first and last lanes correspond to a radiolabeled DNA ladder with known fragment sizes labeled (New England Biosciences N3032). Primers and expected prodcuts are illustrated in Figure 2 **b)** Same as a) but with NIH cohort samples.

**Supplementary Figure 9: Splice graphs at variants with greatest predicted effect on *CYP11B1* splicing.** a) rs6395 is located within the polypyrimidine tract upstream of the 3’ alternative splice site event at exon 9. The T → G transversion disrupts the 3’ splice site consensus sequence and results in a lower MaxEntScan score (MES) as well as lower inclusion of the longer exon 9 (red).  b) rs7818826 is at the first position in cassette exon 4. The A → G transition increases 3’ splice site strength for exon 4, as reflected by the increased MaxEntScan score and evidenced by higher inclusion of exon 4 (orange). This variant was also associated with higher levels of intron 3 retention (green). Colored text communicates the and MAJIQ E[PSI] for the like-colored junction among GTEx v8 donors homozygous for the indicated allele.

**Supplementary Figure 10. Flow chart of the performed analyses**

**FASTA File: *CYP11B1* isoform amino acids sequences**. File containing FASTA format for amino acid sequences for annotated and putative *CYP11B1* protein isoforms. This includes sequences for the threefull length, annotated Uniprot isoforms shown in **Supplementary Figure 6** (P15538, P15538-2, and Q4VAR0) in addition to several putative isoforms generated by alternative splicing labeled as altP15538 followed by the exonic or intronic region that differs from the canonical sequence (i.e. +/- exon 2; +intron3; +/- exon 4; exon9short or exon9long; +/- exon 10).

**Supplementary Methods**

We tested 1,357 variants near the *CYP11B1* locus for predicted splicing effect using the ENSEMBL VEP on GRCh38. Default parameters were used to run VEP via the web interface, with MaxEntScan analysis enabled. MaxEntScan uses a position weight matrix (PWM) model representing the splice site consensus sequences to quantify the strength of a 5’ or 3’ splice site. VEP considers all SNPs within the appropriate consensus motif (5’SS: last 3 bases of exon → first 6 bases of intron; 3’SS: last 20 bases of intron → first 3 bases of exon) for annotated exon boundaries. The reported score is the change in entropy resulting from changing the reference allele at each variant to its recorded alternative allele. A positive score indicates that the reference allele reduces splice site utilization over the alternative allele at that site.

In all, 107 SNPs were tested for any effect on *CYP11B1* itself. Of these, six variants were within splice site consensus motifs at annotated exon boundaries and were therefore tested by MaxEntScan. These variants are, in order from greatest to least effect magnitude (with allelic variants on the sense (-) strand):

- rs6395: T→G, intron upstream of long exon 9, diff=1.236 (R^2^ with rs6410 = 0.83)
- rs7818826: A→G, first position of exon 4, diff=-0.636 (R^2^ with rs6410 = 0.76)
- rs61752786: C→A, 3’ end of exon 7, diff=-0.478 (not in 1000G reference panel; low MAF)
- rs4535: C→T, intron upstream of exon 7 diff=0.394 (monoallelic in CEU population; low MAF)
- rs28418310: C→G, intron upstream of long exon 9, diff=0.364 (R^2^ with rs6410 = 0.0167; low MAF)
- rs5295: G→C, intron upstream of exon 11, diff=-0.285 (R^2^ with rs6410 = 0.09)

The top two variants passed the MAF frequency required by the sQTL analysis (MAF≥0.1) and showed LD with rs6410. The other four varaints were not considered further due to low MAF, low LD with rs6410, and/or not being able to establish the degree of LD with rs6410 from the 1000G reference panel.
